# Machine learning analysis of patients’ perceptions towards generic medication in Greece: a survey-based study

**DOI:** 10.3389/fdsfr.2024.1363794

**Published:** 2024-03-20

**Authors:** Konstantinos Kassandros, Evridiki Saranti, Evropi Misailidou, Theodora-Aiketerini Tsiggou, Eleftheria Sissiou, George Kolios, Theodoros Constantinides, Christos Kontogiorgis

**Affiliations:** ^1^ Laboratory of Hygiene and Environmental Protection, Department of Medicine, Democritus University of Thrace, Alexandroupolis, Greece; ^2^ Laboratory of Pharmacology, Department of Medicine, Democritus University of Thrace, Alexandroupolis, Greece

**Keywords:** generic medication, Greece, machine learning, healthcare, patients’ perception, questionnaire

## Abstract

**Introduction:**

This survey-based study investigates Greek patients’ perceptions and attitudes towards generic drugs, aiming to identify factors influencing the acceptance and market penetration of generics in Greece. Despite the acknowledged cost-saving potential of generic medication, skepticism among patients remains a barrier to their widespread adoption.

**Methods:**

Between February 2017 and June 2021, a mixed-methods approach was employed, combining descriptive statistics with advanced machine learning models (Logistic Regression, Support Vector Machine, Random Forest, Gradient Boosting, and XGBoost) to analyze responses from 2,617 adult participants. The study focused on optimizing these models through extensive hyperparameter tuning to predict patient willingness to switch to a generic medication.

**Results:**

The analysis revealed healthcare providers as the primary information source about generics for patients. Significant differences in perceptions were observed across demographic groups, with machine learning models successfully identifying key predictors for the acceptance of generic drugs, including patient knowledge and healthcare professional influence. The Random Forest model demonstrated the highest accuracy and was selected as the most suitable for this dataset.

**Discussion:**

The findings underscore the critical role of informed healthcare providers in influencing patient attitudes towards generics. Despite the study’s focus on Greece, the insights have broader implications for enhancing generic drug acceptance globally. Limitations include reliance on convenience sampling and self-reported data, suggesting caution in generalizing results.

## 1 Introduction

Generic medicines have worldwide acceptance and utilization due to their benefit on both patients and healthcare systems (Dunne, 2016; Arcaro et al., 2021). In many countries, such as Greece, which have faced economic challenges impacting several sectors, including healthcare, the adaptation of generic drugs was suggested as cost-saving solution (Geitona et al., 2006; Vandoros and Stargardt, 2013; Eger and Mahlich, 2014; Skaltsas and Vasileiou, 2015). In 2019, a report published by Organization for Economic Co-operation and Development (OECD) highlighted various trends in generic medicines adoption across Europe. Greece generic market penetration is relatively low (25.9% in volume) in respect to EU average and other European countries (OECD. Health at a Glance, 2019). Germany and the United Kingdom were noted for their high generic market penetration rates, at 83.3% and 78.5%, respectively. In contrast, Mediterranean countries such as Italy and Spain demonstrated significantly lower penetration rates, with Italy at 38.1% and Spain at 49.3% (OECD. Health at a Glance, 2019).

In Greece, the legislative framework for generic drugs, as outlined in article 11, paragraph 2(b) of the Joint Ministerial Decision No D. YG3α/G.P. 32221/2013, mandates that generic medications must demonstrate bioequivalence to their branded counterparts (Ministry of Health, 2014). This legislation underscores the government’s commitment to integrating generic medicines into the healthcare system. Despite these legislative efforts, the path towards their widespread adoption encounters several obstacles. Challenges such as limited knowledge and misconceptions about generics can be found both among patients and healthcare professionals (Tina et al., 2012; Elpiniki et al., 2013; Labiris et al., 2015; Skaltsas and Vasileiou, 2015; Balasopoulos et al., 2017). In order to increase the acceptance and the daily usage of generic drugs the various misconception should be addressed by promoting better understanding of the benefits that generics can provide to healthcare systems (Colgan et al., 2015).

This study tends to close that knowledge gap by investigating patients’ knowledge and attitudes towards generic drugs. Additionally, the study incorporates a machine learning (ML) model in order to analyze further the factors that influence generic market penetration thus enhancing the knowledge around those findings.

## 2 Materials and methods

### 2.1 Study design and setting

This study was conducted in form of a survey in various cities in Greece from February 2017 to June 2021.

### 2.2 Sample size and eligibility criteria

The study consisted of 2,617 adult patients who were all 18 years old or above. People who were illiterate in Greek, mentally incapable or unwilling to participate were excluded from the study.

### 2.3 Questionnaire

The questionnaire was developed based on relevant literature and was tailored to the characteristics of the Greek healthcare system ensuring its appropriateness for the study’s aim. Key references that help of the survey include works by Shrank et al. (2009) on patient perceptions of generics, as well as studies by Dunne, (2016) and Colgan et al. (2015) highlighting the impact of healthcare provider guidance on patient acceptance of generic medications (Shrank et al., 2009; Colgan et al., 2015; Dunne, 2016).

The questionnaire was divided in three parts each targeting different aspects of the research objectives. Part A was focused on collecting participants’ demographic data (gender, age, employment status and education level) and consisted of four questions. In part B, consisted of 2 questions, participants were asked about their general knowledge of generic medicines, including their understanding of the term generic drug, with options ranging from ‘identical to brand-name drugs but cheaper’ to ‘do not know what generic drugs are.’ This part also asked participants on their sources of information regarding generic drugs, offering choices like ‘books/magazines’, ‘healthcare professionals’, ‘television/radio’, ‘family/friends’, and ‘internet.’. Part C, consisted of 5 questions, aimed at assessing patients’ perception concerning generic drugs investigating the perceived safety of generics, their willingness to switch to generic medication, and the reasons behind their preferences or hesitations, such as concerns over side effects or beliefs about the efficacy of generics compared to brand-name drugs. In total, the questionnaire featured 12 questions. A maximum timeframe of 30 min for completion was given.

### 2.4 Data collection

For the data collection various community pharmacies all over Greece were utilized, benefiting from the country’s diverse geographical landscape. This approach provided a geographically advantageous setting for the research. Participants were approached through a collaborative effort with trained pharmacists. Νo incentives were provided for participation.

A member of the research group was available nearby to clarify any potential misunderstandings and offer detailed explanations. A Participants’ Consent Form and a Participant’s Information sheet were provided outlining the survey’s voluntary nature of the survey in the study and objectives. Upon completion, participants were afforded a cooling-off period to address any concerns before being debriefed by the researcher.

### 2.5 Ethics approval and consent to participate

The study was conducted in accordance with the 1964 Helsinki declaration and its later amendments or comparable ethical standards. The pharmacists’ consent was obtained to recruit participants in the respective community pharmacies. The Ethics and Scientific Committee of Democritus University of Thrace has been approved the study according to the newest decision (D.U.Th./ΕΗ∆Ε/34242/220, 24/02/2023) reassuring that no sensitive or personal information was collected, and the data can be published. Participants were informed about the study, and they could participate anonymously and voluntarily.

### 2.6 Data analysis

Demographic data of participants and all other variables were analyzed using descriptive statistics and the results presented as percentages. Associations between variables were investigated using simple logistic regression. The statistical analysis of independent variables included gender, age, educational level and employment status. The age variable was transformed into a binary variable as either under or over the age of 60. Also, educational level, it was converted into a binary variable separating participants as either high school or lower graduates, or as having education from a higher educational institute (university) or above. Dependent variables, after appropriate data processing, turned into dichotomous, with results presented as odds ratios (OR) with 95% confidence intervals (CI). All analyses were performed with the IBM SPSS statistical software version 22.0 (SPSS Inc., Chicago, IL, United States).

### 2.7 Data acquisition and processing for the ML models

During the process of data acquisition and preprocessing for the ML models, the collected data were classified into two types: categorical and continuous variables. Categorical variables included employment status, education level, knowledge regarding generic drugs, preferred source of information and gender of the participants. Continuous variables, on the other hand included age and year of the survey. During the preprocessing phase, these the two categories were processed distinctly. For continuous variables, missing values were handled by imputing the mean followed by standardization to ensure comparability across different scales. Categorical variables were handled by assigning a placeholder (“missing”) for any gaps and then, one-hot encoding was applied to transform them into a format suitable for the analysis. The target variable, which captured participants’ willingness to switch to generic, was encoded using Label Encoding from the sklearn. preprocessing library.

All the analysis were handled in Google colab, using Python 3.7. The libraries that were deployed were Pandas (version 1.1.5), Scikit-learn (version 0.22.2), XGBoost (version 0.90), Matplotlib (version 3.2.2) and Numpy (version 1.26).

### 2.8 Model development and hyperparameter tuning

For the model development five machine learning models were used: Logistic Regression (LR), Support Vector Machine (SVM), Random Forest (RF), Gradient Boosting (GB), and XGBoost (XGB). Those models were selected for their strength in handling both linear relationships and complex, non-linear interactions inherent in patients’ perceptions towards generic drugs. LR is used for classification tasks, offering a straightforward interpretation of how each variable affects the odds of particular outcome. SVM can find the optimal parameters that separates different classes in the feature space. RF aggregates decisions from multiple decision trees, reduces the risk of overfitting and improves model generalizability. GB and XGB, both gradient boosting models, incrementally build an ensemble of weak models, typically decision trees, to produce a robust predictor.

To optimize these models, hyperparameter tuning process was employed. Hyperparameters are the configurable settings of the model that must be determined before the model begins learning from the data. Unlike model parameters, which are learned directly from the training data, hyperparameters are set in advance to guide the learning process. The method used was GridSearchCV. Grid search involves systematically exploring a range of hyperparameter values by training models on various combinations of these settings, ensuring that the best settings are not overlooked due to manual selection biases. The objective is to identify the combination that produces the most accurate predictions on the data. This methodical approach determines the optimal conditions for model training. Table 1 presents the optimization strategies and hyperparameter tuning for the machine learning models used in this study.

**TABLE 1:** Optimization and Hyperparameter Tuning for Machine Learning Models.

Model	Hyperparameter	Description
LR	Regularization Strength (C)	Adjusted to maintain model simplicity and generalizability by penalizing larger coefficients
	Solver	Optimized for efficient convergence
	Penalty	Specified to complement the data structure
	L1 Ratio	Balanced for Elastic Net regularization, crucial for feature selection
	Constant (Bias/Intercept)	Determined by data centrality to enhance accuracy
SVM	Regularization Parameter (C)	Balances the trade-off between minimizing training data error and model complexity
	Gamma Value	Defines the influence of individual training samples, critical for non-linear kernels
	Kernel Type	Chosen based on the data’s pattern and distribution
	Degree (Polynomial Kernels)	Affects the curvature of the decision boundary
	Coef0	Adjusts the model’s flexibility in higher-dimensional spaces
RF	Number of Trees (N Estimators)	Crucial for the model’s capacity to understand data complexity
	Max Depth	Controlled to prevent overfitting and ensure optimal complexity
	Min Samples Split and Leaf	Determines sensitivity to data variance
	Max Features	Pivotal for diversity within the model ensemble, enhancing prediction accuracy
GB and XGB	Learning Rate	Modulates the contribution of each tree, affecting performance
	Number of Estimators	Controls the sequential trees’ contribution towards the model
	Max Depth	Limited to control tree complexity
	Min Child Weight (XGB)	Tuned to prevent overfitting, enhancing robustness
	Subsample	Optimized to combat overfitting
	Col Sample Bytree (XGB)	Specifically tuned to prevent overfitting, optimizing performance

### 2.9 Training and evaluation

For the training purpose, the dataset was separated training (80%) and testing (20%) sets, to ensure a representative distribution. The training set was used to adjust the models’ parameters until they can accurately predict the outcome variable based on the input. The testing set was then employed to evaluate the models’ predictive performance. After training every model was evaluated regarding their performance by using various metrics such as accuracy, precision, recall, and F1-score. Accuracy provided a broad view of the models’ overall performance by measuring the ratio of correct predictions to total predictions. Precision offered insight into the models’ ability to correctly identify positive outcomes without being overshadowed by false positives, while recall assessed the models’ effectiveness in capturing all relevant instances. The F1-score harmonized precision and recall into a singular metric, presenting a balanced view of the models’ proficiency in identifying true positives amidst false positives and negatives. In addition to these metrics, the Receiver Operating Characteristic (ROC) curve and the Area Under the Precision-Recall Curve (AUCPR) were applied as further indications of performance. The ROC curve, by plotting the true positive rate against the false positive rate across various thresholds, revealed the models’ capacity to distinguish between classes, with the area under the curve (AUC) serving as a quantifiable measure of discrimination ability. A higher AUC value indicated superior model performance in differentiating between the classes. The AUCPR, on the other hand, concentrated on the precision-recall balance, especially pertinent in situations of class imbalance. It underscored the models’ aptitude in accurately predicting positive instances, with the curve’s area serving as a testament to the models’ precision and recall efficacy.

### 2.10 Predictive analysis

In order to predict the likelihood of switching to generic medicines a function was created. This function incorporated the demographic data, the knowledge regarding the term generic medicine and the patients’ source of information. The returned prediction was based on the selected model. In order to facilitate that, an interactive user interface was deployed in order to allow the user to input data related to the data mentioned above. This allowed and easier prediction regarding the likelihood of changing.

The complete code and dataset supporting the findings are available on GitHub at https://github.com/Kandor-Ml/Pharmacoepidemiology.git.

## 3 Results

### 3.1 Demographic characteristics of the participants

A total of 2,617 unique adult individuals filled in the questionnaire, with 1,352 (51.3%) being female. The mean age of participants was reported as 50.85 years, with a standard deviation of ± 18.96 years, indicating the variability of age among participants (Table 2). The complete demographic data are presented in Table 2.

**TABLE 2:** Participants’ demographic data.

Gender	Frequency		Percentage (%)
Male	1,265		48.3
Female	1,352		51.7
Participants		Age Mean ± Standard Deviation	
Total		50.85 ± 18.96	
Males		52.37 ± 19.00	
Females		49.43 ± 18.82	
Age	Frequency		Percentage (%)
Under 60	1,692		64.7
Over 60	925		35.3
Education level			
High school or lower graduates	1,312		50.2
Higher educational institute (university) or higher	1,305		49.8
Employment status			
Employed	1,396		53.3
Unemployed	1,221		46.7

### 3.2 Evaluating participants’ knowledge about generic medicines

From the 2,423 participants who responded to the question regarding the term “generic drug” almost half of them (1,104; 45.5%) responded correctly. Out of the rest 515 (21.2%) of the participants were not familiar with the term, while 378 (15.6%) reported generics as “cheap and poor copies”. Participants over the age of 60 had a lower percentage of correct answers (35,2%) compared to participants under the age of 60 who reported a correct answer rate of 51.4% (OR 1.949, 95% CI 1.645–2.311, *p* < 0.0005). Participants with higher educational degree had answered correct at a rate of 55%, while those with lower educational degree had answered correctly at 36.5%. (OR 0.470, 95% CI 0.400–0.553, *p* < 0.0005). Participants that were actively employed correctly answered at a rate of 55.4%, while those who were currently unemployed/retired answered correctly at a lower rate of 34.6% (OR 2.342, 95% CI 1.988–2.759, *p* < 0.0005).

### 3.3 Participants’ perception about generic drugs

A significant portion of the participants (69.8%) expressed a positive inclination towards generics when proposed by their physician/pharmacist. On the other hand, 30.2%, reported a preference for branded drug despite the availability of generics. Attributing their choice to a perceived higher quality (53.7%) and better efficacy (26.8%) associated with branded medications.

**FIGURE 1:**
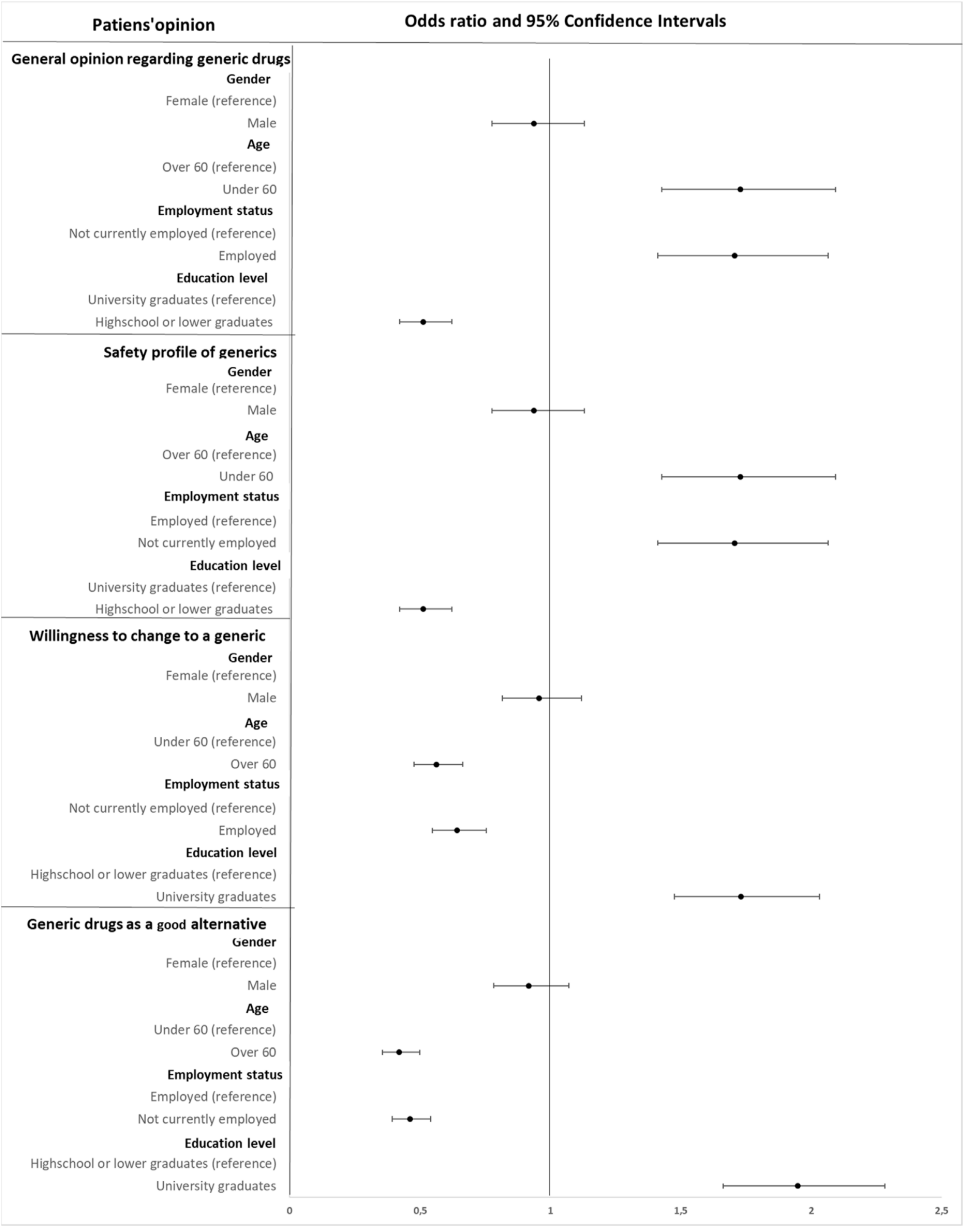
Forest plot displaying the odds ratios and 95% confidence intervals for various factors influencing patients’ opinions about generic drugs. The odds ratios are represented by dots and the horizontal lines through the dots depict the 95% confidence intervals. The vertical line at 1.0 indicates no effect.

In terms of affordability, 86.5% of the participants concurred that generic are a more affordable option. Participants, who were familiar with the term “generic medicine” were more inclined to regard generics as a cosτ-effective alternative (OR 1.588, 95% CI (1.351–1.863), *p* < 0.0005). Furthermore, participants receiving information from physicians/pharmacists were more likely to acknowledge the affordability of generic drugs (OR 1.599, 95% CI (1.367–1.869), *p* < 0.0005) (Figure 1).

### 3.4 Source of information and evaluation

Among the 2,617 participants, the majority (32.6%) identified their physician/pharmacist as the primary source of information with online sources (20.2%), family/friend (17.4%), television/radio (16.3%) and printed press (13.1%) also mentioned as significant information sources. Analysis by genders revealed that both females (34.2%) and males (30.9%) mainly consulted their physician/pharmacist. Participants under the age of 60 preferred their physician/pharmacist (29.9%) slightly more than online sources (28.8%). In contrast, participants over the age of 60, were more inclined to rely on their physician/pharmacist (37.4%), with family and friends (26.3%) and television/radio (23.1%) also serving as key sources. Considering educational background, high school graduates were more likely to turn to family/friends (21.4%) after their physician/pharmacist (35.2%), whereas university graduates more frequently used online sources (23.6%) in addition to their physician/pharmacist (29.9%).

The accuracy of participants’ understanding the term “generic drug” reported their physician/pharmacist as primary source was 75.6%, followed by those relied on printed press (65%). Participants reported family/friends as their primary source had the lowest correct response rate (16.1%) with those relying on media reporting a slightly higher rate (21.7%).

### 3.5 Patients’ opinion regarding generic drugs

The perception of safety associated with generics was mostly positive among the participants (74.9%). Analysis by gender indicated that opinions on the safety of generic were similar between females and males, with 77.3% of females and 78.4% of males agreeing on their safety (OR 0.936, 95% CI (0.775–1.130), *p* < 0.0005). Participants over 60 considered generics to be safe by 71.4% (OR 1.728, 95% CI (1.426–2.094), *p* < 0.0005). Employed participants considered generics safe by 82.1% (OR 1.707, 95% CI (1.412–2.064), *p* < 0.0005). Education, also was linked to safety perceptions, with 72% of participants who were high school graduates considered generics safe (OR 0.512, 95% CI (0.422–0.621), *p* < 0.0005) (Figure 1).

### 3.6 Branded substitution

In the context of substituting branded drugs with generic alternatives, 58.7% of participants expressed willingness to make the switch. Both genders seemed positively inclined to switch their branded medication (59.2% of females and 58.1% of males) (OR 0.955, 95% CI (0.815–1.119), *p* < 0.0005). However, participants aged over 60 showed less willingness for switching to generics, with only 49.5% in favor (OR 0.562, 95% CI (0.476–0.664), *p* < 0.0005). Employed participants were more positive to changing to generics (63.7%) compared to unemployed participants (52.9%) (OR 0.642, 95% CI (0.547–0.753), *p* < 0.0005). High school graduates seemed indecisive regarding the generic substitution (51.9%) compared to university graduates (65.2%) (OR 1.730, 95% CI (1.474–2.031), *p* < 0.0005) (Figure 1).

The primary motivation for those willing to switch was the recommendation of their physician (66.2%), followed by advice from their pharmacist (13.5%), and the lower cost of generics (13.5%). Women were more likely to follow a physician’s advice (68.4%) than men (64.3%). Trust in physicians was higher among older participants (74.1%) compared to younger ones (63.3%). Participants with lower educational levels expressed greater trust in physicians (68.7% *versus* 64.7%). Conversely, among participants hesitant to switch to generics, 24.1% doubted their efficacy, 25.7% preferred not to change their medication, 29.5% feared potential side effects, and 20.8% prioritized health over cost. Concerns about side effects were a common barrier across both genders and more pronounced among participants over 60 (31.9%). Educational background influenced perceptions as well, with lower-educated participants more concerned about side effects (34%) and higher-educated participants more wary of changes in their treatment regimen (28.3%). Also, participant with lower educational level considered generic drugs different from branded and were less willing to change their medication (OR 1.730, CI (1.474–2.031), *p* < 0.0005).

Overall, women had a slightly more favorable view of generics, (50.8% *versus* 48.6%) (OR 0.916, 95% CI (0.783–1.071), *p* < 0.0005). Participants over 60 viewed generics less favorably than younger individuals (35.9% *versus* 57.1%) (OR 0.420, 95% CI (0.355–0.498), *p* < 0.0005). Unemployed and retired participants showed more positively towards generics (39.5%) (OR 0.461, 95% CI (0.393–0.541), *p* < 0.0005) while those with lower education were more cautious, perceiving generic drug use as potentially dangerous (58.7%) (OR 1.948, 95% CI (1.662–2.283), *p* < 0.0005) (Figure 1).

### 3.7 Model hyperparameters

Throughout the development of the ML models various parameters were evaluated following trials and with multiple combinations of those parameters. The final parameters selected are detailed in Table 3.

**TABLE 3:** Hyperparameter values across all models.

Model type	Hyperparameters	Values
LR	C, Solver, Penalty, L1 Ratio, Fit Intercept	10, liblinear, l2, 0.2, True
SVM	C, Gamma, Kernel, Degree, Coef0	0.1, 0.1, poly, 2, 1.0
RF	N Estimators, Max Depth, Min Samples Split, Min Samples Leaf, Max Features	10, 20, 10, 4, auto
GB	N Estimators, Learning Rate, Max Depth, Min Samples Split, Min Samples Leaf, Max Features, Subsample	100, 0.1, 3, 2, 1, sqrt, 1.0
XGB	N Estimators, Learning Rate, Max Depth, Min Child Weight, Subsample, Colsample Bytree	100, 0.01, 3, 1, 1.0, 0.7

Based on that configuration, LR model used a regularized linear approach C with an L2 penalty to optimize for moderate complexity and prevent overfitting. SVM is configured with a polynomial kernel, in order to capture non-linear patterns for relations that are not strictly linear. RF was designed with a limited number of trees and depth to avoid overfitting. GB model aimed to detect detailed patterns withing the data. Similarly, XGB focused on capturing detailed patterns, but utilized slower learning rate, potentially leading to a refined model that captures subtle patterns in the data.

### 3.8 Model performance

In the analysis of the predictive models, the evaluation was focus on accuracy, precision, recall, and the F1-score for each class. LR showed the second highest accuracy of 75.39%. It exhibited a very high predictive capability for Class 1 with precision 0.73, recall of 0.91 and F1-score 0.81 and bit lower precision for Class 0 (0.82). The accuracy of RF is the highest of all (76.19%) with good precision and F1-scores in both classes. SVM and GB exhibited similar accuracy (74.60%). XGB also showed good performance with accuracy 73.41%. The full report of the metrics is mentioned on Table 4.

**TABLE 4:** Model performance metrics.

Model	Accuracy (%)	Precision class 0	Recall class 0	F1-score class 0	Precision class 1	Recall class 1	F1-score class 1
LR	75.39	0.82	0.55	0.66	0.73	0.91	0.81
SVM	74.60	0.83	0.51	0.63	0.72	0.92	0.81
RF	76.19	0.82	0.56	0.67	0.74	0.91	0.81
GB	74.60	0.80	0.55	0.65	0.72	0.90	0.80
XGB	73.41	0.87	0.45	0.59	0.70	0.95	0.80

**FIGURE 2:**
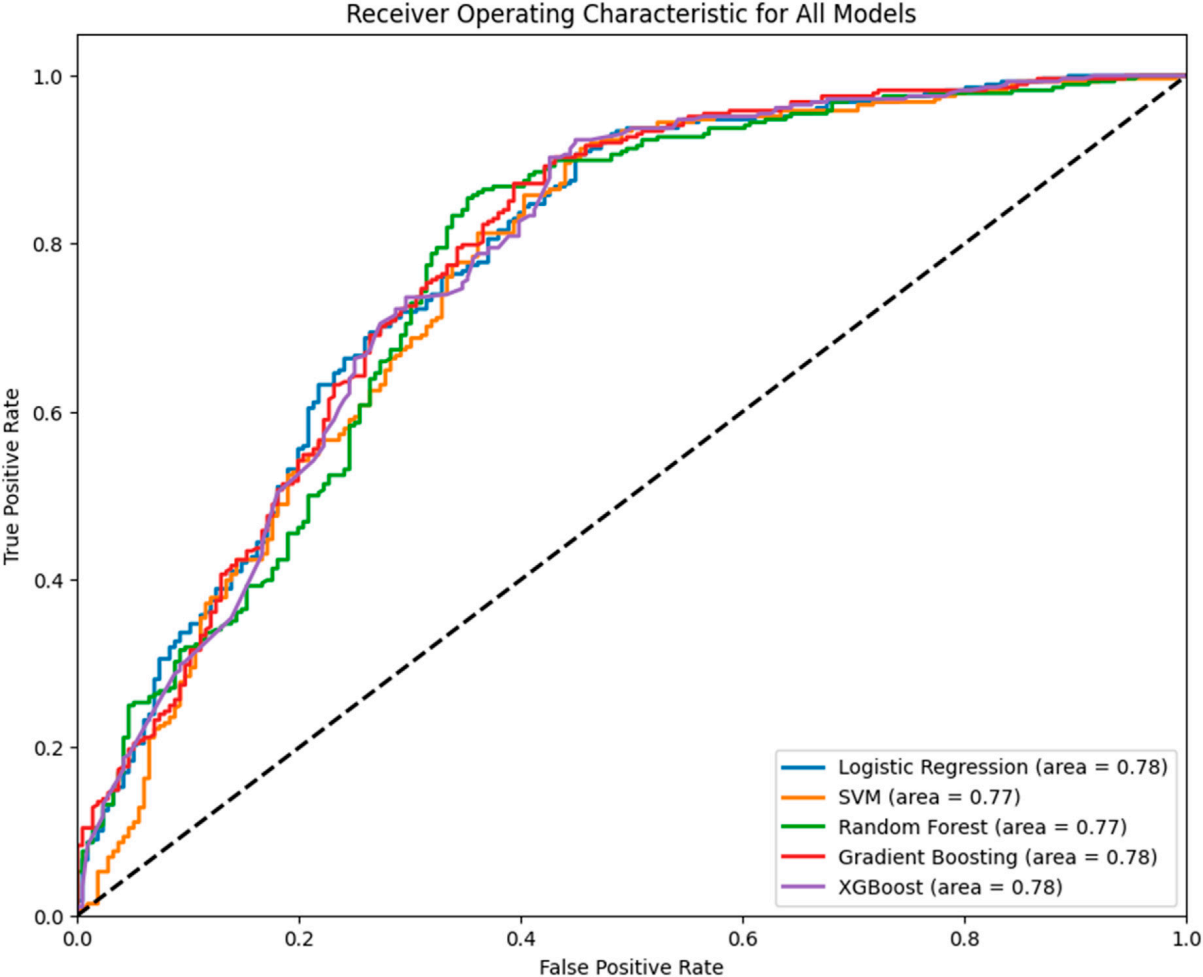
Receiver Operating Characteristic (ROC) curves for five distinct predictive models along with its area under the curve (AUC) score (AUC-ROC). The *x*-axis represents the false positive rate, while the *y*-axis shows the true positive rate. The diagonal dashed line indicates the performance of a random classifier.

**FIGURE 3:**
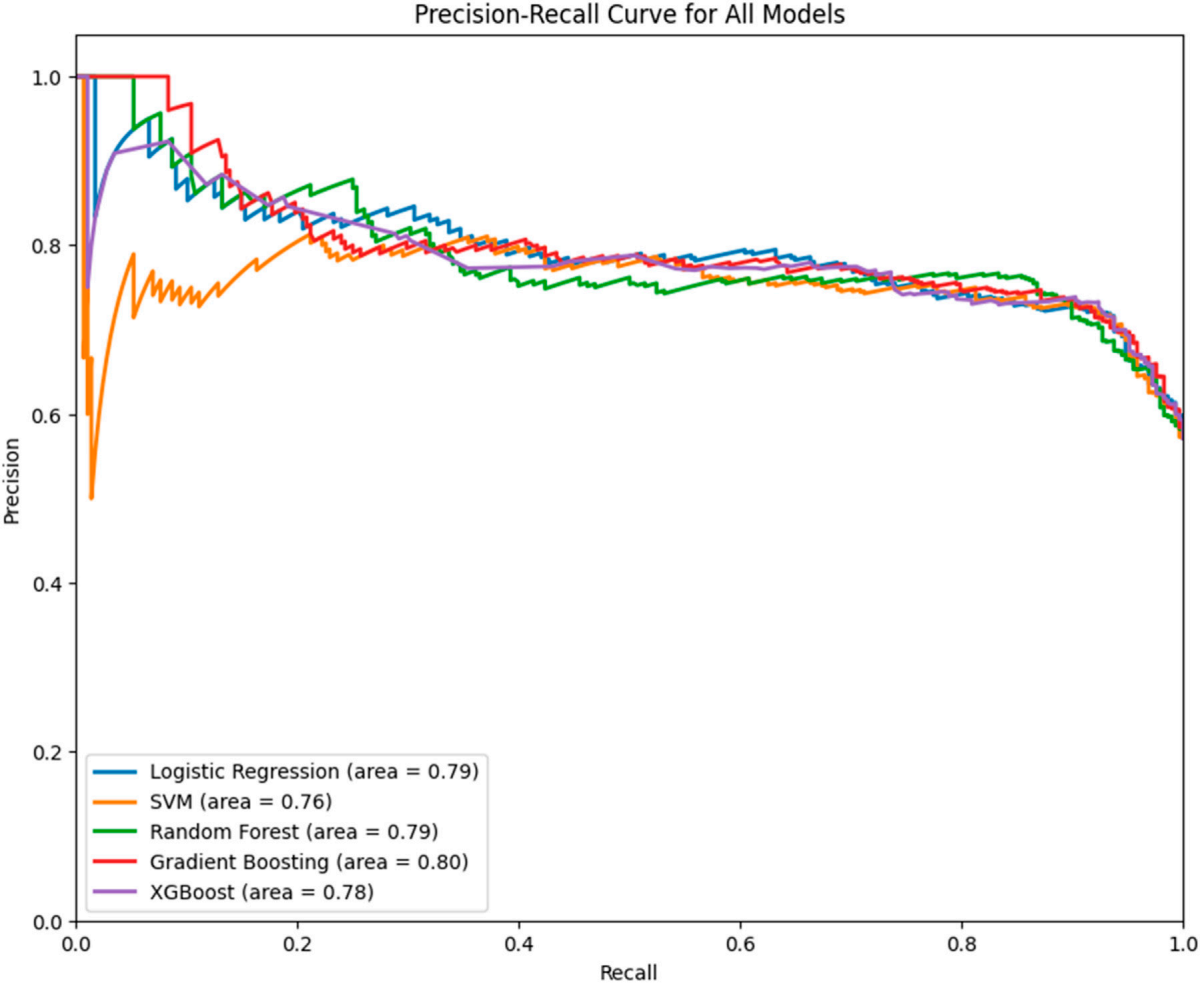
Precision-Recall Curve for five different predictive models and its corresponding area under the curve (AUC) score (AUCPR). The *x*-axis indicates the recall, and the *y*-axis represents precision.

Since accuracy as metric is not sufficient for model evaluation ROC and Precision-Recall curve were used. The Area Under Curve (AUC-ROC) was used to quantify the model ability to differentiate between classes and Area Under Curve for Precision-Recall (AUCPR) to address any potential imbalance to the dataset (Figure 2, 3).

In evaluating the performance of the various predictive models, both the LR and RF models demonstrated strong capabilities. LR provided high precision, recall, and F1-scores, particularly for Class 1 predictions, indicating a strong ability to identify the positive class correctly. RF yielded the highest overall accuracy and maintained good precision and F1-scores for both classes, suggesting robust predictive power. While LR offers the advantage of interpretability, which is crucial in clinical applications for understanding the influence of each variable, RF’s higher accuracy and consistent performance across different metrics indicate its superior predictive ability for this dataset. Considering these aspects, despite the strong individual class performance of LR, the RF model is selected as the most appropriate for this study due to its balance of accuracy, reliability, and performance consistency as evidenced in the ROC and Precision-Recall curves. This model’s ability to handle the dataset’s categorical and continuous features effectively, without overfitting, further supports its selection. Finally, while XGB is considered high performance model, its effectiveness seems to be limited in that dataset probably due to the mix of categorical variables and sampling methods.

### 3.9 Key predictors

The investigation into the factors influencing patients’ willingness to switch to generic medication, conducted through LR, SVM, RF, GB and XGB models, has provided substantial evidence on the predictors impacting such decisions.

LR highlighted the important role of patients’ perceptions and understanding of generics, identifying beliefs about the equivalency and cost-effectiveness of generics, as well as the influence of healthcare-related education and information from healthcare professionals, as key predictors. SVM highlighted the significance of patients’ perceptions and knowledge about generics. RF brought additional dimensions into focus, such as the impact of age and specific beliefs about generics. It was observed that misconceptions about generics being cheap and poor copies deter patients from switching, whereas recognizing generics as equivalent to brand-name drugs encourages the switch. GB and XGB mentioned as key predictors the misconceptions and the beneficial influence of accurate knowledge on the decision to switch to generics. XGB, in particular, emphasized the important role of healthcare professionals as a primary source of information.

Building on the predictive strengths identified in the models, the following section highlights their practical utility through hypothetical scenarios, demonstrating the models’ applicability in real-world settings.

### 3.10 Practical application of predictive modeling

To demonstrate the practical utility of predictive modeling, hypothetical scenarios involving two patients were introduced, designed to assess their changing willingness to switch to generic medicines over time. The selected model based on performance was RF. Patient X is a 60-year-old retiree in 2023 who lacks knowledge about generic drugs and uses media, family/friends and physician/pharmacist for information. The scenario explores how shifts in source of information and evolving understanding of generics, can influence decision making among the elderly. Patient Y is 23-year-old female. Y has not yet graduated by the year 2023 but gradually get her MSc. The target is to explore the perception shifting on various educational levels. Regarding her employment status in order to create a realistic scenario she starts as unemployed, passes to private sector and keeps going to public in order to aim for her PhD. By that, various employment status on younger participants is explored. Finally, based on the analysis performed which suggest university graduates are more informed about generics Y is familiar with the term. Finally, her change of source of information is realistic since she gets professionally active and begins to trust healthcare providers more. All the results are exhibited on Table 5.

**TABLE 5:** Model predictions for the two hypothetical patients.

Year	Age	Gender	Education level	Employment status	Knowledge about generic drugs	Source of information	Likelihood of changing to generic medicines
X (2023)	60	Male	High School Graduate	Retired	Does not know what generic drugs are	Media	No
X (2024)	61	Male	High School Graduate	Retired	Cheap and bad copy of brand drugs	Friends/Family	No
X (2025)	62	Male	High School Graduate	Retired	Same as brand but cheaper	Friends/Family	No
X (2026)	63	Male	High School Graduate	Retired	Same as brand but cheaper	Printed press/books	No
X (2027)	64	Male	High School Graduate	Retired	Same as brand but cheaper	Doctor/Pharmacist	Yes
Y (2023)	23	Female	High School Graduate	Unemployed	Cheap and bad copy of brand drugs	Online	No
Y (2024)	24	Female	University Graduate	Private Sector	Same as brand but cheaper	Online	Yes
Y (2025)	25	Female	University Graduate	Private Sector	Same as brand but cheaper	Doctor/Pharmacist	Yes
Y (2026)	26	Female	Msc/PhD	Private Sector	Same as brand but cheaper	Doctor/Pharmacist	Yes
Y (2027)	27	Female	Msc/PhD	Public Sector	Same as brand but cheaper	Doctor/Pharmacist	Yes

## 4 Discussion

This study explores the perceptions of Greek patients’ regarding the substitution of generic drugs for brand-name medications. A significant portion of participants indicated a preference for receiving information from their healthcare provider (Sewell et al., 2011; Mishuk et al., 2018). However, a significant number of the participants still expressed a preference for brand-name medicines. This can be attributed the general misconception which suggests that brand-name are superior in quality and efficacy (Shrank et al., 2009; Colgan et al., 2015; Skaltsas and Vasileiou, 2015). This misconception is often rooted in subjective beliefs, rather than empirical data (Shrank et al., 2009).

The impact of demographic factors on participants’ perceptions was explored further in the study. It was observed that older participants and participants with educational level showed reduced awareness and understanding of generics (Iosifescu et al., 2008). This observation aligns with the finding of Iosifescu et al., who mentioned that different demographic groups, display varied levels of knowledge about generics particularly the elderly, who demonstrated limited awareness (Iosifescu et al., 2008). In addition, previous studies indicate that factors such as education and age significantly influence patients’ perspectives towards generics (Choudhry et al., 2016; Mishuk et al., 2018).

This study also revealed healthcare providers as the primary source of information in shaping participants’ attitudes towards generics. Participants who were informed by their physicians or pharmacists showed better understanding and a more favorable opinion towards generics (Colgan et al., 2015; Dunne, 2016). This highlights the crucial role that healthcare providers in patients’ education about generics, as mention by Colgan et al. (Colgan et al., 2015). Trust in the advice of physicians advice was identified as a predominant factor influencing patients’ willingness to switch to generic drugs (Palagyi and Lassanova, 2008), thus highlighting further the important role of healthcare providers in shaping patients’ perceptions regarding medication choice.

The integration of ML models is increasingly recognized for its significance in various healthcare tasks as highlighted by the study conducted by Rajkomar et al. (Rajkomar et al., 2019). Artificial Intelligence (AI) models also show great potential in enhancing patient outcomes (Topol, 2019). The application of ML algorithms is particularly effective in analyzing heterogenous dataset, such as surveys, providing deeper insights from patents’ responses and augmenting the understanding of complex behavioral patterns and perceptions. A study by Bari et al. demonstrated the utility of the RF algorithm in examining patients’ interactions with healthcare providers providing insights in patients behavioral traits (Bari et al., 2020). This aligns with the current study’s implementation of RF model in order to achieve a better understanding of similar behavioral traits on a survey-based dataset. MacNell et al., in 2023 in their study employed GB model and National Health and Nutrition Examination Survey dataset highlighting the importance of sampling weights for creating generalizable predictions (MacNell et al., 2023). Meanwhile, in a study outside of healthcare sector but with a similar dataset, Ramosaco et al. used LR to analyze the performance levels of university freshmen highlighting the versatility of LR in demographic based datasets (Ramosaco et al., 2015). Both of those studies justify the application of GB and LR algorithms in the current study. The notable underperformance of XGB, could be related to the challenges associated with imbalanced dataset. In a study conducted by Velarde et al., where XGB’s performance was evaluated across different dataset sizes and class distributions, was revealed that detection performance improves with increased data volume but decreases with data imbalance (Velarde et al., 2023).

This study highlights the importance of various factors in augmenting generics market penetration in Greece. The statistical analysis highlights the importance of healthcare providers in influencing patients’ perceptions, a finding that aligns with earlier studies (Shrank et al., 2009; Topol, 2019). Given the impact on patients’ perceptions it is critical for governments to invest in continuous education for healthcare professionals. Thus, educational programs can help to correct any potential misconception around generic medications, thus, thereby creating a more informed patient base. Additionally, launching awareness campaigns, especially aimed at elderly and patients with lower educational levels of education (Iosifescu et al., 2008; Palagyi and Lassanova, 2008; Tsiantou et al., 2009). Finally, the use of ML and AI models can enhance predictive capabilities by providing a deeper understanding of datasets, which in turn facilitates the creation of more targeted interventions to boost the market penetration of generics (Rajkomar et al., 2019; Topol, 2019). The implementation of those measures can contribute to creating cost-saving strategies for healthcare systems.

Regarding the study’s limitations, the use of convenience sampling method and reliance on self-reported data are noted as potential biases. Also, the applicability of those findings, is primarily referred to Greece’s taking healthcare system, which may limit their worldwide application. Additionally, while ML demonstrates potential accuracy predicting various outcomes, factors such data leakage, the necessity of improved accuracy and the risk of overfitting remain relevant issues (Steyerberg and Vergouwe, 2014; Gianfrancesco et al., 2018; Shortliffe and Sepúlveda, 2018; Wiens et al., 2019).

In conclusion, this study highlights the importance of physician and pharmacists in shaping patients’ perceptions around generic drugs, thereby augmenting generic market penetration. The statistical analysis, also, highlights the most misinformed demographic groups, an insight that could prove critical for governments in the development of healthcare strategies. The employment of ML models, and RF in particular, expands the dataset by offering predictions for various target groups, thereby enhancing the understanding of the factors influencing patients’ decision. This model’s predictive accuracy offers a foundation for developing communication strategies for promoting generics adaptation. However, it is important to address models’ limitations including risks of data leakage, for the need for improved accuracy and the potential of overfitting. Through refinement of these models and strategic application, central governments can more effectively help the adoption of generic medications.

## Data Availability

The original contributions presented in the study are included in the article/Supplementary Material, further inquiries can be directed to the corresponding author.
